# Focus on the Outer Membrane Factor OprM, the Forgotten Player from Efflux Pumps Assemblies

**DOI:** 10.3390/antibiotics4040544

**Published:** 2015-11-12

**Authors:** Gilles Phan, Martin Picard, Isabelle Broutin

**Affiliations:** Laboratoire de Cristallographie et RMN Biologiques, CNRS UMR 8015, Faculté de Pharmacie, Université Paris Descartes, 75006 Paris, France; E-Mails: gilles.phan@parisdescartes.fr (G.P.); martin.picard@parisdescartes.fr (M.P.)

**Keywords:** antibiotic resistance, *Pseudomonas aeruginosa*, OMF membrane protein, 3D structure

## Abstract

Antibiotics have been used extensively during several decades and we are now facing the emergence of multidrug resistant strains. It has become a major public concern, urging the need to discover new strategies to combat them. Among the different ways used by bacteria to resist antibiotics, the active efflux is one of the main mechanisms. In Gram-negative bacteria the efflux pumps are comprised of three components forming a long edifice crossing the complete cell wall from the inside to the outside of the cell. Blocking these pumps would permit the restoration of the effectiveness of the current antibiotherapy which is why it is important to increase our knowledge on the different proteins involved in these complexes. A tremendous number of experiments have been performed on the inner membrane protein AcrB from *Escherichia coli* and, to a lesser extent, the protein partners forming the AcrAB-TolC pump, but less information is available concerning the efflux pumps from other virulent Gram-negative bacteria. The present review will focus on the OprM outer membrane protein from the MexAB-OprM pump of *Pseudomonas aeruginosa*, highlighting similarities and differences compare to the archetypal AcrAB-TolC in terms of structure, function, and assembly properties.

## 1. Introduction

After several decades of intensive antibiotherapy we are now facing the advent of multi-resistant strains from several species [1]. Among them there is *Pseudomonas aeruginosa*, an opportunistic Gram-negative pathogen responsible for numerous nosocomial diseases. It is referred by the Infectious Diseases Society of America as one of the “ESKAPE” bugs *(Enterococcus faecium*, *Staphylococcus aureus*, *Klebsiella pneumoniae*, *Acinetobacter baumanni*, *Pseudomonas aeruginosa*, and *Enterobacter species)*, that is to say the main group of multi-resistant bacteria responsible for serious hospital infections [2]. It develops mainly in immune-compromised individuals such as patients suffering of cystic fibrosis, cancer, HIV or burn victims. Without being as deadly as *Clostridium difficile*, *Staphylococcus aureus* or *Streptococcus pneumoniae* [3], *P. aeruginosa* is responsible for around 10% of incurable nosocomial infections. It is naturally resistant to antibiotics due to a highly impermeable outer membrane. On top of that, a synergy between a low-level expression of the porins and an increase of the efflux pump’s activity leads to a very efficient expulsion of the antibiotics outside of the cell, even before the drugs could reach the target [4,5,6]. Efflux pumps are polyspecific transporters that increase the acquisition of multidrug resistance (MDR) due to the recognition and the transport of a broad range of substrates from all antibiotic families [7]. It has been demonstrated that the inhibition of these MDR pumps increases antibiotic susceptibility [8] and reduces the probability of emergence of antibiotic resistant mutants [9], making them promising targets for drug design. In Gram-negative bacteria, these efflux pumps are organized as multicomponent systems [10], formed with transporters from different families like the ABC (ATP-binding Cassette) [11], the MFS (Major Facilitator Superfamily) [12], and the RND (Resistance Nodulation Cell Division) [13]. For the latter, transport is made possible by the reversible assembly of a tripartite protein complex consisting of: (i) a membrane protein of the RND family embedded in the inner membrane, responsible for the active transport (energized by the proton motive force generated upon proton counter-transport); (ii) a periplasmic protein, fixed to the inner membrane by an N-terminal palmitoyl anchor whose putative role is to stabilize the whole complex and; (iii) an exit channel from the OMF family (Outer Membrane Factor). The sequencing of the entire genome of *P. aeruginosa* allowed the identification of 12 pumps, eight of which (MexAB, MexCD, MexEF, MexHI, MexJK, MexXY, MexVW, and TriABC) are involved in antibiotic resistance [8,14,15,16,17,18,19,20,21,22]. The eight pumps display very different resistance phenotypes and only the MexAB-OprM system is expressed constitutively, whereas the others are expressed under special circumstances. These pumps confer resistance to most β-lactams including fourth generation cephalosporins, quinolones, aminoglycosides, trimethoprim-sulphamides, tetracycline, chloramphenicol, erythromycin, and triclosan. Only the pump MexXY-OprM is able to efflux aminoglycosides [23].

In order to restore the use of current antibiotic molecules, one of the envisaged approaches is to block the assembly of the three proteins forming these efflux pump edifices. However, this is possible only if a precise structural knowledge of the complex formation and that of each protein partner are available. Most of the structural information obtained on efflux pumps from the RND family came from the study of the AcrAB-TolC pump from *E. coli* [9,24,25,26,27,28,29] (with 43 deposited structures for AcrB in the Protein Data Bank (PDB)), even though the structures of the three isolated proteins from the MexAB-OprM pump from *Pseudomonas aeruginosa* were solved [30,31,32,33,34,35], as well as the heavy-metal efflux pump CusBA-CusC from *E. coli* [36,37,38]. In addition we can find in the PDB the structures of isolated members of efflux pumps from other bacterial species, like the OMFs MtrE (*Neisseria gonorrhoeae*) [39], CmeC (*Campylobacter jejuni*) [40] and VceC (*Vibrio cholerae*) [41], the MFP ZneB (heavy-metal efflux adaptor from *Cupriavidus metallidurans*) [42], and the RNDs MtrD (*Neisseria gonorrhoeae*) [43] and ZneA (*Cupriavidus metallidurans*) [44]. Among these different proteins, AcrB has been extensively studied and reviewed in order to understand the mechanism linking the antibiotic’s efflux to the pump assembly and the proton transfer. The different MFPs also bring a lot of attention (for review, see [45]) but surprisingly much less the OMFs, which are generally considered as passive holes in the outer membrane. In this short review will be gathered and analysed the different specific knowledge published on the OprM channel from *Pseudomonas aeruginosa*, which belongs to the TolC family, but shows some particular and interesting structural features.

## 2. Structural Aspects

### 2.1. Description of the OprM Solved Structures

**Figure 1:**
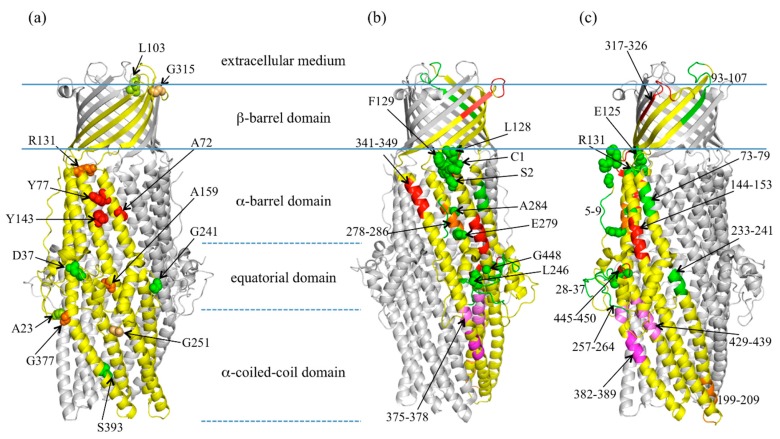
OprM structure with reported mutagenesis studies (**a**) the point of insertion of the malarial epitope performed by Wong [50] are presented in balls of different colors from red to green following the functional consequence on resistance. Red: protein not expressed or with resistance comparable to the OprM-inactivated strain; green: protein as active as the wild type (wtOprM); (**b** and **c**) two different views of OprM structure with mutations and deletions reported in [51] (same colour code as in (**a**)) and deletion studied in [50,52] that lead to important functional modifications when compared with wtOprM (colored in magenta).

The different OMF structures that have been solved share similar folding despite a low percentage of sequence identity (e.g., 21% between TolC and OprM with only 40% of similarity). The channel is formed by three monomers, each of which carries an internal duplicated structure, consisting of two α-helices and two β-strands. One of the helices is interrupted in the middle by a region called the equatorial domain (Figure 1), which is made of three small helices and an unstructured loop, forming a buoy around the helical barrel that extends over 100 Å in the periplasm. This hinge zone gives a full flexibility to the protein that probably contributes to the opening and the assembly of the channel. The membrane embedded part of the trimer consists of a barrel of β-sheets with a thickness of about 40 Å across the outer membrane. The overall structures of the known OMF proteins are very similar but some differences exist, mostly between TolC and OprM. The N-terminal portion of OprM is much longer, adding a short extra helix in the buoy and a palmitylated N-terminal cysteine [46,47] anchored to the outer membrane. In TolC, the C-terminus is 45 residues longer and displays an additional β-sheet in the equatorial domain, which seems to be important for the efflux function [48]. Finally, the extracellular loop between the third and the fourth β-strands of the porin domain is longer in TolC. The periplasmic domain involved in the interactions with the MFP and RND partners, is located under the buoy region and is called “α-coiled-coil” domain. This area consists of a ring of six α-helices with three internal helices surrounded by a larger second ring. The first structure of OprM [34] was solved in a space group where the three monomers are linked by a pure crystallographic symmetry (R32) and, therefore, gives no clue about a possible asymmetric structure behind the efflux mechanism since the monomers are constrained to be identical. Nevertheless, we later solved OprM structure in the P2_1_2_1_2_1_ space group which allows us to visualize a whole trimer [35]. The three monomers of OprM structure show few differences that are mainly localized on the exposed amino acid side chains. Among them, there are R194 and R405 that were identified as two key residues because the formation of a chimeric complex TolC-MexAB was possible upon mutation of these equivalent residues of TolC to OprM sequence [49]. Nevertheless, the symmetrical structure of OprM seems to indicate that the asymmetric movement observed for the RND partner [27,32] is not transmitted to the channel, at least when isolated from the complex.

### 2.2. Highlight on Hotspot Residues

As the first OprM structure [34] was solved four years after the TolC one [28], several models were built for OprM, based on the sequence similarity with TolC, and the accuracy of the models were evaluated by different mutation and/or deletion experiments performed on the *oprM* gene. The expression level and functionality of each construct were analyzed by MICs measurements performed with OprM-inactivated strains [50,51,52] and, for some of them, expression experiments were realized in *E. coli* strains with *OmpC*, *OmpF*, and *PhoE* deleted [50]. Once the crystal structure of OprM was solved, some of these genetic modifications could be correctly mapped out, thus revealing some hot spots that are mandatory for the function or the stability of OprM. In a first approach, a random insertion of the malarial epitope (NANP) repeats led to thirteen different mutants [50], among which six insertion sites resulted in a non-translated, non-inserted or degraded protein when produced in a *Pseudomonas* strain, and only three of them were not detectable by Western blot when produced in *E. coli*. Interestingly, the three insertion sites leading to divergent results between the two bacteria (R131, A159, G377) (Figure 1a) correspond to a specific region of OprM when we superpose the TolC structure. In OprM, R131 interacts with the extended N-terminus and A159 is closed to the small α-helix 39–44 that is not found in TolC. Similarly, G377 is localized in a loop in OprM, whereas an additional β-strand is found in TolC, forming a beta-sheet with its elongated C-terminus. These observations show that the sites are essential and specific to OprM and that only when expressed in *P. aeruginosa*. Thus studying efflux pump function, interactions or expression must always be crosschecked in the putative organism. Concerning the seven other insertion sites, the resulting mutated proteins are properly expressed and show variable functional perturbation as measured by MIC experiments [52]. The most perturbing modifications are insertions at G251 localized in the buoy and at G315 in one of the extra-cellular loops (Figure 1a) that was not predictable as these residues are located in flexible loops at the surface of the protein structure. In the other way around, the effect of five different deletions was analyzed by the same method [52]. The ∆29–36 and the ∆93–100 deletion (Figure 1b) resulted in almost no difference in antibiotic resistance compared to the wild type OprM (wtOprM). On the contrary, ∆375–378, ∆429–439 and, above all, ∆382–389 presented a large impact on the protein function (Figure 1c). Those three regions are all located in the periplamic α-coiled-coil domain, which is supposed to open in an iris-like movement for drug expulsion. It has to be noted that insertion at position S393 was not very disturbing while the deletion of 382–389 in the same helix almost abrogates OprM recruitment. This seems to pinpoint an interacting interface localized on the solvent-exposed surface of the α-helices rather than a direct assembly with the tip of the α-coiled-coil domain.

Other series of mutations or deletions focused on OprM regions that are well conserved within the OMF homologues in *P. aeruginosa*, *P. putida*, *B. cepacia*, and *S. maltophilia*, were analyzed by MIC measurements in different strains [51]. None of the constructs harboring the deletion of the highly-conserved C-terminal motif ^446^LGGGW^450^ shows OprM expression. Nevertheless, single mutations within the motif or deletion of the C-terminus sequence downstream did not affect OprM expression and the protein was totally functional. The same results were reported for another conserved motif ^125^ELDLFGR^131^ structurally closed to the N-terminus. These two conserved motifs probably correspond to a strategic structural role common to all the OMF. The first one (446–450) corresponds to the end of the last helix at the buoy level and represents the last folded part of the protein. The second one (125–131) corresponds to a hinge region between the β-barrel domain and the α-helical domain. In addition it is in close contact with the N-terminus of OprM, adding additional stability to the N-terminus extension. On the contrary, the deletion of another motif ^278^AEHQLMAAN^286^ including several highly-conserved amino acids leading to an expressed, but not functional, protein. Interestingly, single mutations of the motif did not affect OprM function. The same group made some other deletions, which led to mutants with different antibiotic resistance profiles (Figure 1b,c). Altogether, it appears that the conserved sequences of the OMF could play different roles, not only a simple structural stability of the channel, but also a functional assembly or gating.

## 3. Role of OprM in the Assembly with the Other Protein Partners

### 3.1. Structural Aspects

**Figure 2:**
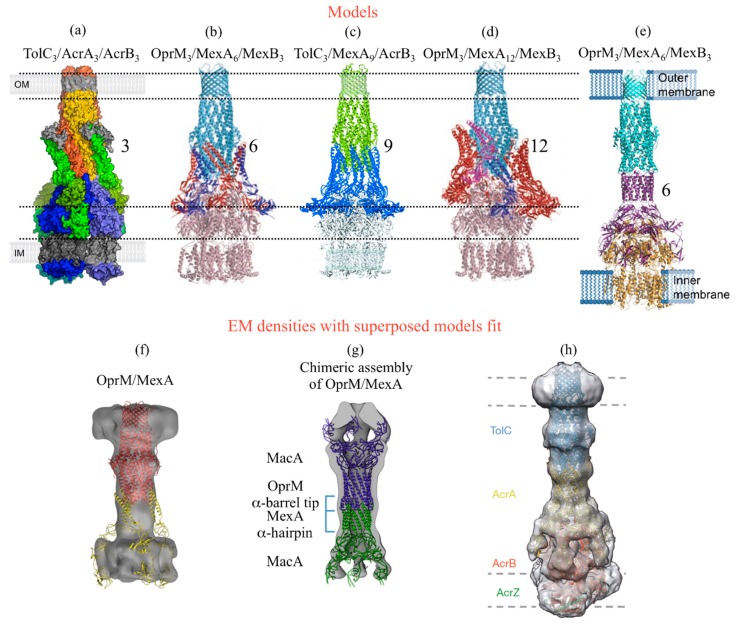
Published models and EM densities of the efflux pump assembly involving OprM or its *E. coli* counterpart TolC. (**a**) Surface representation of a proposed 3:3:3 assembly model for the TolC-AcrA-AcrB pump from *E. coli* coloured in orange, green and blue respectively (reproduced with permission from Symmons M.F. *et al.*, Proceedings of the National Academy of Sciences [55]; published by The National Academy of Sciences, 2009). (**b** and **d**) Cartoon representations of the respectively proposed 3:6:3 and 3:12:3 assembly models for the MexA-MexB-OprM pump from *P. aeruginosa.* OprM and MexB are coloured light-blue and pink respectively. Monomers of MexA are in blue, red or magenta (reproduced with permission from Akama H. *et al.*, Journal of Biological Chemistry [30]; published by *The American Society for Biochemistry and Molecular Biology* ©2004). (**c**) Cartoon representation of the proposed 3:9:3 assembly model of the chimeric TolC-MexA-AcrB pump, respectively coloured in green, blue and light-blue, based on the fit of their respective 3D structure. (reproduced with permission from Higgins M.K. *et al.*, Proceedings of the National Academy of Sciences [31] “National Academy of Sciences, U.S.A ©2004”). (**e**) Cartoon representation of a proposed 3:6:3 assembly model for the OprM-MexA-MexB pump from *P. aeruginosa*, respectively coloured in cyan, purple and orange (reproduced with permission from Xu Y. *et al.*, Journal of Biological Chemistry [70] published by *The American Society for Biochemistry and Molecular Biology* ©2011*).* (**f**) Electronic microscopy (EM) density for the *P. aeruginosa* OprM-MexA complex reconstituted into proteoliposomes, with a proposed 3:3 model equivalent to the one presented in (**a**) (reproduced with permission from Trépout S. *et al.*, Biochem. Biophys. Acta [67]; published by Elsevier B.V. 2010). (**g**) EM density of a chimeric assembly of MacA (blue) in which the α-coiled-coil domains were replaced by those of OprM (purple) or MexA (yellow), together with the cartoon representation of the corresponding fitted model (reproduced with permission from Xu Y. *et al.*, Journal of Biological Chemistry [71]; published by *The American Society for Biochemistry and Molecular Biology* ©2012)*.* (**h**) EM density of a stabilised assembly of the *E. coli* TolC-AcrA-AcrB pump in complex with AcrZ together with the cartoon representation of the corresponding fitted model, respectively coloured in blue, yellow, red and green (reproduced with permission from Du D. *et al.*, Nature [57]; published by Macmillan Publishers Ltd Copyright © 2014). The different models published before 2011 (**a**–**d**) were based on the hypothesis of a direct interaction between the RND and the OMF proteins, surrounded of variable oligomeric states of the MFP partner. On the contrary, all the EM densities are in favor of a tip-to-tip interaction between the MFP and the OMF. We can see in (**f**) the discrepancy between the two hypothesis, as the MexA structure positioned as in (**a**) does not fit with the OprM/MexA EM density.

There has been a long controversy about the oligomerization state of the MFP protein in the pump edifice, and various models of the entire pump assembly have been proposed for AcrAB-TolC and MexAB-OprM (Figure 2). However, each proposed model was consistent with a three-fold symmetry for each partner. Indeed, MexA structures seem to indicate that it is possible to make a full ring by juxtaposing nine MexA molecules [31]. It has even been suggested that 12 MexA could surround the OprM-MexB complex [30]. This would require adaptability of the various domains of MexA with respect to each other, which is possible thanks to the high flexibility of the protein [53]. MexA is, therefore, prone to adapt easily to the structure of its partner, in particular the peristaltic movement of MexB. However, models attempting to dock MexA in the periplasmic pocket of MexB have led to a more modest degree of oligomerization of six [30] or even three MexA [54,55] per OprM/MexB trimer. Then the question remained unclear, until two experimental structures of the complex were solved: the crystallographic structure of the CusAB complex [56] showing six CusB molecules around the CusA trimer, and the structure of the AcrAB-TolC-AcrZ complex by electron microscopy [57]. Nevertheless the latter result comes from a chimeric construct that constrains the oligomeric state of each partner and, thus, cannot be taken as a definitive model of the pump assembly. Some original approaches such as blue native gel electrophoresis [58] or Fluorescence Recovery After fringe Pattern Photobleaching (FRAPP) [59] gives some interesting insights on that assembly question. The first approach proved that MexA needs to be pre-dimerized to interact with OprM and that depends on the palmitoylation of its N-terminus residue, even though it has been shown that MexA does not need this post-translational lipidation to be functional *in vivo* [60]. The second approach showed that the oligomerization state of MexA is pH dependent, being of six under pH 6.7 and two above pH 7.2. This idea of MFP functioning as a dimer has been reinforced recently by the description of the TriABC-OpmH pump of *P. aeruginosa*, which needs two distinct MFPs having different role for the function [61]. All the results converge to a 3:6:3 edifice, but this assembly seems to be variable. It raises the question whether the pump is permanently assembled and opens only when necessary or whether it is formed only under specific conditions as it has been suggested recently by studies performed on reconstituted proteins into liposomes [62,63]. This is still an open question that will probably need more efforts to be answered.

A second question about the pump assembly also stirred up controversy of whether the OMF protein interacts with the MFP or directly with its RND partner. A recent *in silico* approach [64] analyzed the consistency between the two hypotheses and the currently available data, and concluded that both models possibly exist. No crystallographic structure has ever been solved yet of a complex involving an OMF with one of its partner, which seems to indicate that these complexes are not stable enough for the crystallization process, even if the interaction between MexA and OprM has been proved to be detectable *in vitro* in the absence of MexB by blue native polyacrylamide gel electrophoresis (BN-PAGE) [58] on proteins purified in detergent, by FRAPP [59] and by cryo-EM on proteins inserted in an artificial membrane [65], and *in vivo* by pull-down experiments [66]. At present, all the structural information available on this aspect has been recorded by electronic microscopy. The first MexA-OprM model has been obtained by reconstitution of the proteins in proteoliposomes [67]. The electronic density was clearly in favor of an elongated assembly, *i.e.*, a tip-to-tip manner between the different partners, instead of an interaction of the MFP coiled-coil tips directly on the equatorial domain of the OMF as it was generally admitted before [30]. Nevertheless this elongated model was observed on different chimeric complexes involving the protein MacA, an adaptor MFP protein working with an ABC transporter [68], in which the coiled-coil tips were replaced by those of the proteins of interest. The chimeric constructs showed that TolC interacts with MacA [69] or AcrA [70], just as OprM with MexA [71], in a similar tip-to-tip manner involving a trimer of the OMF protein and a hexamer of the MFP one. This particular elongated assembly was recently confirmed by the resolution of the cryo-EM structure of the chimeric complete AcrAB-TolC-AcrZ assembly [57]. The equivalent complex for MexAB-OprM has not been solved yet, but one can suppose it will form a similar assembly.

### 3.2. Mutational Analysis

Apart from the structural approach, many mutagenesis, protein cross-reactivity, and complementation studies have been initiated to understand the assembly of the three proteins.

**Figure 3:**
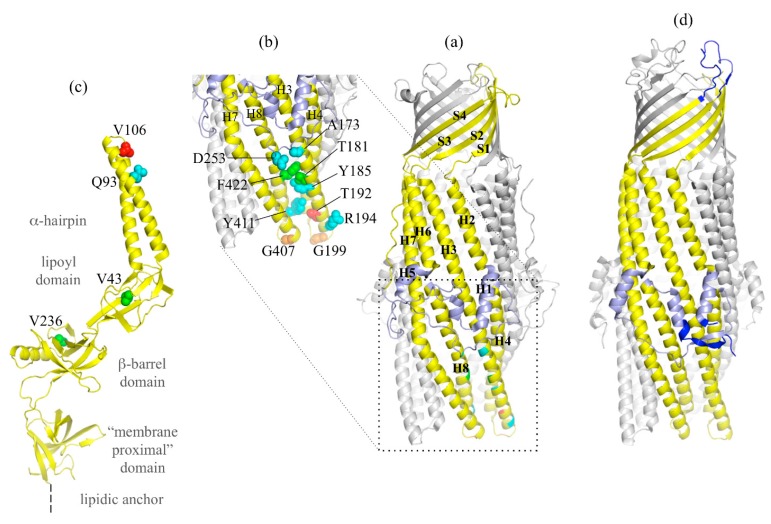
OprM structure with reported gain of function mutants and α-coiled-coil domain mutations. (**a**) Cartoon representation of the OprM trimer with two monomers colored in grey and one monomer colored in yellow with the equatorial domain in light blue. The secondary structure adopted is indicated. (**b**) Zoom of the OprM α-coiled-coil domain. Residues colored in blue (A173, Y185, D253, Y411, and R194) correspond to the equivalent positions of the TolC mutants making TolC functional with MexAB. Residues colored in green (T181I and F422I) correspond to the OprM mutations suppressing the MexA-V106M loss of function. The residue colored in red (T192) corresponds to a loss of expression of OprM. Residues colored in orange (G199A and G407A) correspond to non-functional mutants. (**c**) Cartoon representation of one monomer of the MexA structure (PDB code 2V4D) [55] with the three residues cited in the text presented in sphere. V106, whose mutation in methionine leads to a non-functional MexAB-OprM pump, is in red. V43 and V236, whose mutation respectively in methionine and phenylalanine restore the loss of function of MexB-G220S, are in green. Q93, whose mutation in arginine makes MexA able to function with OprN, is in blue. (**d**) Cartoon representation of TolC for comparison with the same color code as in OprM, with the exception of the three regions largely different from OprM structure (N-terminus, extracellular loop and C-terminus), colored in blue.

The different OMFs have different specificities with respect to their cognate pumps (Table 1). In *P. aeruginosa* OprM is not only the exit duct of MexAB [72], but also of MexXY [8,73,74] and MexJK [75]. In addition, it can replace OprJ with MexCD [76] and OprN in MexEF [77] without affecting the substrate’s elution profile of these pumps. Conversely, OprN is only able to interact with MexEF [78], but when trying to interchange the MFPs, OprM is not able to assemble with MexB and MexX, neither does MexY with MexA according to the pull-down experiments [66]. Swapping the hairpin domain of MexE into MexA results in a chimeric MFP that works with OprM-MexF, but not with OprN-MexF. Moreover, a single mutation in MexA (Q93R, see Figure 3) makes it functional with OprN-MexB, but not as efficient as the native OprM-MexAB [79]. The other way round, MexA bearing the hairpin from MexE does not form a functional pump with OprM-MexB [79]. This is in accordance with a central role of the MFP in the assembly. Chimeric OMFs, replacing each half of OprM with the equivalent OprN region (OprMN and OprNM), were also analyzed for their ability to work with their cognate partners, respectively MexAB and MexEF [80]. Surprisingly, both constructs were functional with MexEF but none of them restored the antibiotic resistance level of the native MexAB. In *E. coli*, OprM is able to interact with the AcrAB multi-drug transporter, as proved by cross-linking experiments, but the pump is not functional [81]. Chimeric constructs, mixing the MexB sequence into AcrB, showed that only the first 60 amino acids of MexB, involved in the trimer contacts, can be modified without affecting the pump function [81]. Concerning the MFP, replacing the AcrA hairpin domain by the one of MexA in the OprM-AcrAB assembly restores the functionality [82]. In contrast, TolC does not interact with MexAB, but can work with AcrA-MexB [83]. OprM [84] and TolC [85] are able to interact with VceAB resulting in a functional pump but, conversely, VceC cannot functionally replace TolC, although the chimeric assembly is still possible [85]. OprM is no more functional with VceAB when its 20 C-terminal residues are truncated [84]. The C-terminal tail of OprM has already been showed to be involved in the interaction with MexAB [51]. Nevertheless, when a modified VceA mutated in the hairpin domain (VceA^D155Y^) is used instead of the wild type, the function with OprM-∆20Cter [84] is restored. In addition, when replacing the C-terminal tail located after the sequence ^444^KALG^447^, by the one of VceC or TolC, the chimeric OprM is still able to function with MexAB and VceAB [84].

All of these results highlight particular areas of the proteins involved in specific recognition between the different pumps, but some of the results have to be taken with caution, as they do not always take place in the native bacterial strain. Together with structural data, several models have been suggested for the assembly of the edifice (as previously mentioned). In order to verify the consistency of these models, different mutations were introduced into the sequence of the different partners. As already mentioned, TolC cannot work with MexAB in *E. coli*, unless some specific mutations are introduced [49]. Among 53 analyzed variants, four TolC mutants are able to increase by more than 10-fold the resistance to novobiocin of a ∆AcrAB-TolC *E. coli* strain co-transformed with MexAB (TolC-D121N, I133A, V198D and I369F), and a fifth one (TolC-Q142R) was shown to potentiate the effect of TolC-V198D or TolC-I369F. The equivalent residues in OprM are A173, Y185, D253, Y411, and R194 (Figure 3). Except for the mutant TolC-V198 which is localized in the equatorial domain, the other four mutants belong to the α-coiled-coil domain, suggesting a strong involvement of this region in the interaction with the other proteins partners. It has to be noticed that TolC-I369F is located inside the helices bundle which are supposed to be released for a proper interaction and opening. Another mutational study was performed in *Pseudomonas aeruginosa* in order to highlight the regions important for the function of the MexAB-OprM pump then probably involved in the protein/protein interactions [86]. As MexA-V106M was reported to be non-functional [87], OprM suppressor mutants were identified based on the restoration of carbenicillin resistance phenotype. Two OprM mutants (T181I and F422I) (Figure 3) are localized on the same helix as the TolC mutants that are functional with MexAB. The OprM-T181I mutation even results in an overproduction of the whole pump indicating a probable stabilizing effect of the mutation on the tripartite complex formation. This hypothesis is reinforced by the fact that T181I is also able to restore the function of a MexB-G220S mutant that has been proved to destabilize the MexB trimer [88]. Thus, these different approaches support the implication of residues from helices H3 and H8 in the complete pump assembly. In order to study the role of amino acids localized at the extremity of these helices, in particular the loops linking H3 to H4 and H7 to H8, most of them were replaced by alanine or threonine. Among them, one resulted in an unproduced protein (T192A) and two others (G199A and G407A) abolished the MexAB-OprM function. These glycines are localized at the far extremity of the periplasmic α-coiled-coil domain, suggesting a direct interaction with MexAB. Curiously, a functional pump with both OprM-G199A and OprM-G407A is restored by the MexA mutants V43M and V236F, localized respectively in the lipoyl and β-barrel domains, far from the MexA hairpin. These results proved that the MFP is a major actor in the specificity of interaction between the different pumps.

**Table 1:** Summary of the functional complementarity between different wild-type and mutated proteins forming an efflux pump from different species.

**Antibiotic Resistance Experiments Performed in *Pseudomonas aeruginosa* Strains**																										
OMF	OprM		OprM		OprM		OprM	OprM		OprM	OprM	OprN		OprM		OprN		OprM	OprMN *		OprNM *		OprMN *		OprNM *	
MFP	MexA		MexX		MexX		MexA	MexJ		MexC	MexE	MexA		MexA hairpin/MexE		MexA hairpin/MexE		MexE hairpin/MexA	MexA		MexA		MexE		MexE	
RND	MexB		MexY		MexB		MexY	MexK		MexD	MexF	MexB		MexF		MexF		MexB	MexB		MexB		MexF		MexF	
Functionality	Yes		Yes		No		No	Yes		Yes	Yes	No		Yes		No		No	No		No		Yes		Yes	
References	[66,72,77,80,87]		[8,73]		[66]		[66]	[75]		[76]	[77,79]	[77]		[79]		[79]		[79]	[80]		[80]		[80]		[80]	
**Antibiotic Resistance Experiments Performed in *E. coli* Strains**																										
OMF	OprM	OprM		OprM		OprM			OprM				OprM		TolC		TolC			TolC		TolC		TolC		TolC
MFP	MexA	MexX		AcrA		MexA			MexA hairpin/AcrA				VceA		AcrA		MexA hairpin/AcrA			MexA		VceA		MexC		MexX
RND	MexB	MexY		AcrB		AcrB60/MexB			AcrB				VceB		MexB		AcrB			MexB		VceB		MexD		MexY
Cross-linked evidenced assembly formation	Yes	No		Yes		Yes			Yes				Yes		Yes		Yes			No		Yes		Yes		Yes
Functionality	Yes	Yes		No		Yes			Yes				Yes		Yes		Yes			No		Yes		Yes		Yes
reference	[89]	[74]		[81,82]		[81]			[82]				[84]		[83]		[82]			[83,85]		[85]		[85]		[85]

* OprNM corresponds to the N-terminal 1–266 amino acids (aa) of OprN and the C-terminal 274–468 aa of OprM with no intervening gap. OprMN corresponds to the N-terminal half of OprM (1–275 aa) and the Cterminal half of OprN (269–447 aa) with no intervening gap.

All of these results highlight particular areas of the proteins involved in specific recognition between the different pumps, but some of the results have to be taken with caution, as they do not always take place in the native bacterial strain. Together with structural data, several models have been suggested for the assembly of the edifice (as previously mentioned). In order to verify the consistency of these models, different mutations were introduced into the sequence of the different partners. As already mentioned, TolC cannot work with MexAB in *E. coli*, unless some specific mutations are introduced [49]. Among 53 analyzed variants, four TolC mutants are able to increase by more than 10-fold the resistance to novobiocin of a ∆AcrAB-TolC *E. coli* strain co-transformed with MexAB (TolC-D121N, I133A, V198D and I369F), and a fifth one (TolC-Q142R) was shown to potentiate the effect of TolC-V198D or TolC-I369F. The equivalent residues in OprM are A173, Y185, D253, Y411, and R194 (Figure 3). Except for the mutant TolC-V198 which is localized in the equatorial domain, the other four mutants belong to the α-coiled-coil domain, suggesting a strong involvement of this region in the interaction with the other proteins partners. It has to be noticed that TolC-I369F is located inside the helices bundle which are supposed to be released for a proper interaction and opening. Another mutational study was performed in *Pseudomonas aeruginosa* in order to highlight the regions important for the function of the MexAB-OprM pump then probably involved in the protein/protein interactions [86]. As MexA-V106M was reported to be non-functional [87], OprM suppressor mutants were identified based on the restoration of carbenicillin resistance phenotype. Two OprM mutants (T181I and F422I) (Figure 3) are localized on the same helix as the TolC mutants that are functional with MexAB. The OprM-T181I mutation even results in an overproduction of the whole pump indicating a probable stabilizing effect of the mutation on the tripartite complex formation. This hypothesis is reinforced by the fact that T181I is also able to restore the function of a MexB-G220S mutant that has been proved to destabilize the MexB trimer [88]. Thus, these different approaches support the implication of residues from helices H3 and H8 in the complete pump assembly. In order to study the role of amino acids localized at the extremity of these helices, in particular the loops linking H3 to H4 and H7 to H8, most of them were replaced by alanine or threonine. Among them, one resulted in an unproduced protein (T192A) and two others (G199A and G407A) abolished the MexAB-OprM function. These glycines are localized at the far extremity of the periplasmic α-coiled-coil domain, suggesting a direct interaction with MexAB. Curiously, a functional pump with both OprM-G199A and OprM-G407A is restored by the MexA mutants V43M and V236F, localized respectively in the lipoyl and β-barrel domains, far from the MexA hairpin. These results proved that the MFP is a major actor in the specificity of interaction between the different pumps.

## 4. The Outflow Mechanism through OprM Channel

Even if a large amount of information ensues from mutagenesis studies, there is no consensus about the opening mechanism. In the various structures of outer membrane proteins, both ends of the pore are generally closed. This is not consistent with their role in the efflux and suggests that they should open in response to a signal most likely triggered by one or two of the other partners. It was suggested for TolC that the porin could be opened following a movement comparable to that of cameras, an “iris-like mechanism” [28]. This would consist in an untwisting of the helix segments of the α-coiled-coil domain. Later, a normal modes analysis of the structure of OprM [35] suggested an opening movement consisting of the combination of twisting and extension of the entire channel, aligning the α-coiled-coil circumference to the ß barrel domain in a breathing motion. This would allow a diameter of about 30 Å along the channel, ending with an opening of the valve formed by the extracellular loops. In addition, this normal mode study has underlined a possible locking role for residues located at the periplasmic end of the pore, forming two points of closure. They involve charged residues that form hydrogen bonds and even some salt bridges. Residues involved in the lock differ from one OMF to another, but they all strengthen the interaction between internal and external helices and/or the three monomers. In order to evaluate the role of these stiffening residues, different mutations were introduced in TolC, breaking one by one the different bonds, and the opening of the resultant protein was evaluated by electrophysiology measurements [90,91] and/or structure determination [29,92,93]. All together these experiments highlighted the major role of the hydrogen bond D153-Y362 from helices H4 and H7 linking the two pairs of helices forming the α-coiled-coil domain of one monomer, and that of the salt bridge D153-R367 from helices H4 and H8 also linking the same pairs of helices but from two different monomers. The other analyzed bonds (Q136-E359 and T152-R367) were proven to be of less importance. The double mutation Y362F-R367S led to an increase by more than 10 times the protein conductance (1000 pS) compared to the wild type (80 pS) [90], with an opening movement limited to the α-coiled-coil helices extremities (2 and 6 Å, respectively, for the inner and the outer helices), as proved by the crystallographic structures [93]. The other way round, some cysteines were introduced at strategic positions in TolC in order to create rigid disulfide bonds to keep the protein closed [91]. *In vivo* experiments measuring the TolC-dependent export of hemolysin, showed a 80% reduction of the transport in spite of proper assembly when helix H4 is cross-linked to helix H7 from a sided monomer, highlighting the importance of the α-coiled-coil domain flexibility. Finally, a Co(NH_3_)${}_{6}^{3+}$ ion, shown to annul the TolC conductance, was co-crystallized with the protein, and was found to be coordinated by the D374 amino acid from the three monomers [92] drawing attention to two aspartate rings (D371 and D374) 5 Å apart from each other. Surprisingly, by mutating each of these aspartic acids, it was shown that both residues are important for interaction, leading to the hypothesis they create a strong electrostatic field, responsible of local binding steps in molecules efflux. In OprM, the equivalent residues are not all conserved. As for the two aspartate rings, only the one corresponding to TolC-D374 exists (OprM-D416), the second one is a threonine in OprM. Nevertheless, it is also involved in an inside constriction close to the extremity of the periplasmic α-coiled-coil domain, by creating three inter-monomer salt bridges with OprM-R419. Among the other salt bridges and hydrogen bonds that are important in TolC opening mechanism, only one is strictly conserved in OprM (TolC-D153-Y362 equivalent to OprM-D205-Y404). However, OprM-D209-R403 can be found instead of TolC-D153-R367, which are both located at the extremity of the periplasmic entrance, even if OprM-D209-R403 links the H7 extremity to H8 and TolC-D153-R367 links H4 to the H8 helix from a side monomer. Finally, at the location of TolC-D371 corresponding to the smallest constriction, we find in OprM a hydrophobic residue creating tight van der Waals interactions (OprM-L412). As for TolC, several attempts to insert OprM in supported lipidic membranes were performed in order to measure the protein conductance leading to comparable results between published values (850 pS [52], 726 pS [94]) for experiments performed in 1M KCl buffer. It has to be noticed these measured conductance are ten times higher than that for TolC, but no explanation was proposed so far.

Another way to study protein movements is to subject their high-resolution structure to molecular dynamic (MD) simulations. Each member of the efflux pump edifice has been studied in that way leading to very interesting hypotheses about the opening and drug pathways through the three proteins (for a review, see [95,96]). Concerning the OMF proteins, wild-type and several mutated TolC were studied using short MD simulations (≈20 ns) [97,98] indicating a large motion of the extra cellular loops, closing and opening over the β-barrel like a lid, suggesting a gate function. However, a longer simulation (≈300 ns) on the wtTolC [99] in the presence of NaCl showed a free motion of opening and closing of the extracellular loops, dismissing this gate hypothesis. On the periplasmic side, an increase of the helices flexibility was observed in TolC-Y362F-R367S (TolC^YFRS^) by Vaccaro *et al.* [97], whereas potassium-dependent opening was found by Schulz *et al.* [98] in TolC-Y362F-R367E (TolC^YFRE^) and Y362F-R367D (TolC^YFRD^). The 300 ns simulation performed on the wtTolC [99] in presence of NaCl confirmed an important role of ions in the opening mechanism, two sodium being found to interact with D371, T366, T368 and D153 all localized in the lowest part of the periplasmic end. The protein was able to open on that side only when the simulation system was emptied from all Na^+^ ions. To go further in the comprehension of the ions’ actions on the periplasmic opening of TolC, six mutants (TolC^YF^, TolC^RE^, TolC^RS^, TolC-D153A (TolC^DA^), TolC^YFRE^, and TolC^YFRS^) were submitted to 60–150 ns MD simulations using a minimal ion concentration [100]. This study permitted the exploration of a larger conformational space ending with an opening three times larger of wtTolC at D374 compared with the solved wtTolC structure. This revealed an asymmetric opening of all the mutants, except TolC^YF^, which had a more constricted periplasmic gate than the wtTolC and was even more stable, suggesting that only the interprotomer interaction R367–D153 is critical for the conformational stability of the periplasmic gate. The effect of pH lowering on the stability of the protein was also analyzed on wtTolC [100], showing the periplasmic gate is much smaller at pH below 5.0 than at pH 7.5, suggesting a pH-dependent conformational change of the pump regulating the assembly and the export.

**Figure 4:**
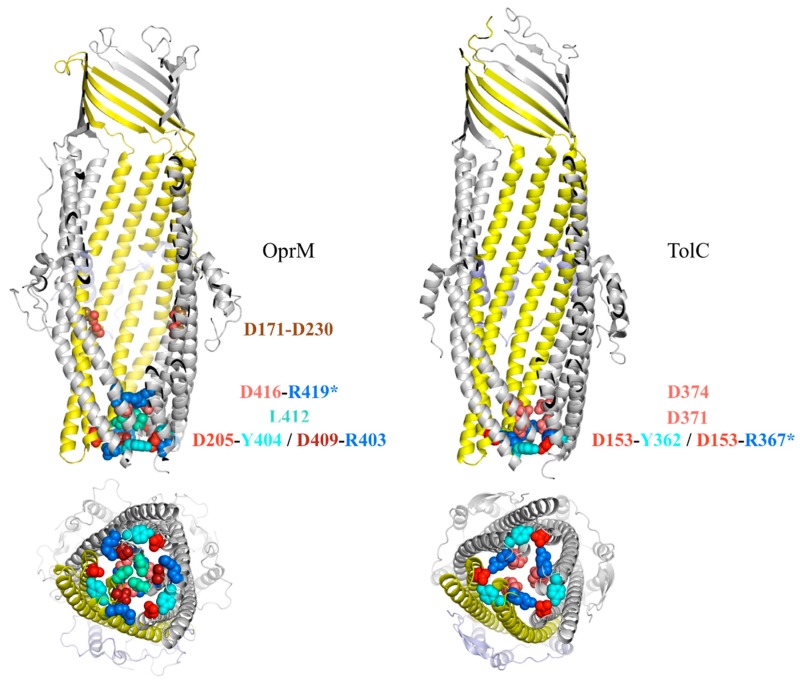
Comparison of the periplasmic domain of OprM and TolC. Amino acids involved in the closing gate are represented in colored balls. When the interaction is inter-monomeric, a star is added on one residue name. The D171-D230 link indicated on OprM corresponds to the sodium binding side highlighted by molecular dynamic simulations [101].

Concerning OprM, a 200 ns dynamic simulation was performed [101] resulting in the same flip-flap movement of the extracellular loops as observed in TolC [99]. On the periplasmic side, even if the movement was similar to TolC, it led to a much more opened protein, which is consistent to a more important value obtained on OprM in electrophysiology measurements [52,94]. Contrarily to TolC, an upper sodium-binding site was found inside the channel at D171-D230 (Figure 4), close to the equatorial domain. Although the function of this binding-site has not been investigated so far, it has to be noted that it is located close to the extended TolC C-terminal that is not present in the shorter sequence of OprM.

## 5. Conclusions

As it is shown in this review, OprM, like its OMF counterparts, cannot be reduced to a passive hole in the outer membrane of Gram-negative bacteria. All the experimental and computational information gathered here indicate that OprM operates in the same way as its homologue TolC, but with some interesting structural specificities. Indeed, mutagenesis analysis demonstrated that the two structures present local differences, mainly in their N- and C-terminus, which would explain the selectivity of the assembly or the drug-efflux phenotype. The opening of the periplasmic entrance of the OMF follows a common untwisting motion of the α-coiled-coil domain. Nevertheless, in the absence of the cognate partners, the resulting opened diameter of OprM is more important than TolC, as highlighted by a 10-times higher conductance measured on membrane embedded system. Residues lining the interior of the α-coiled-coil domain play different roles in both proteins, by not linking the same helices within the trimer and by creating different repartition of charges inside the tunnel, which can be important for the selection of the expulsed molecules and the efflux efficiency. As for TolC, OprM interacts with two other partners to form the complete efflux system via its α-coiled-coil domain. The interacting zone is supposed to be the tips of the helices forming this domain, even if some mutagenesis data does not favor this hypothesis, giving some grey zones about the opening mechanism. OprM is able to function with a large panel of pumps and the MFP’s hairpin seems to be determinant in the selectivity of the assembly partners. At present, the functional oligomeric association of the complete efflux system RND-MFP-OMF is likely to be 3:6:3, and the MFP seems to pre-dimerize to interact with the others upon acidic pH. Whether the assembly pre-exists or not in the absence of the substrate is still an open question that requires further investigation. Despite the fact that OMFs are not usually targeted in drug design to combat antibiotic resistance, keeping OprM either in a closed state or away from assembling would certainly block the drug efflux in *Pseudomonas aeruginosa* and that of several pumps at the same time. An interesting approach limiting OprM expression by targeting directly its mRNA [102] proved to be efficient to increase the sensibility of *Pseudomonas* strains to the five most commonly used families of antibiotics. That is why OprM should be explored more in the field of anti-pseudomonas therapy.
